# Extraction of a Piece of Lead-pencil from the Bladder by Perineal Incision

**Published:** 1857-05

**Authors:** S. D. Gross

**Affiliations:** Prof. of Surgery in Jefferson Medical College, Philadelphia


ART. IV.—*Extraction of a Piece of Lead-Pencil from the Bladder by a small Incision through the left side of the Perinæum.* By S. D. GROSS, M.D., Professor of Surgery in the Jefferson Medical College.

THE subject, Philip Deyman, a farmer, thirty-nine years of age, of Guelph Township, Wellington County, Canada West, had been troubled for some time with an obstruction to the free passage of his urine, arising, as he supposed, from "gravel." To "remove the gravel," as he expressed it, he was in the habit of introducing into his urethra a piece of common lead pencil, which enabled him to urinate with greater ease. A retention of urine having existed for upwards of twenty hours, on the 6th of February,

1857, he resorted to the same procedure, but the pencil escaped from his grasp, and was drawn into the bladder. In a few days it gave rise to severe pain, and a constant desire to micturate; the pain was very acute just after the act; and at no time could he retain his water longer than an hour. The other symptoms were, itching in the head of the penis; an increased discharge of mucus; slight scalding in passing his urine; and occasionally interruption of the stream. The patient reached Philadelphia on the 3d March, and presented himself to me the next day, when, by means of a sound, the foreign substance was readily detected. A cathartic was ordered in the evening, and on the following day, an attempt was made to extract the pencil through the urethra; but failing in this, I decided on the lateral operation of lithotomy. On Friday morning, March 6th, the patient being thoroughly under the influence of ether, the operation was performed in the usual manner, except that the incisions were very small. The pencil was found to be behind the pubic symphysis, and was removed without the slightest difficulty. Very little blood was lost; and a grain of morphia was administered soon after the operation. On the sixth day, the urine had ceased to flow from the wound, and up to the time of the patient's departure, it passed entirely through the urethra.

On the 13th of March, the patient had several chills, and the right testicle began to enlarge. These symptoms soon disappeared under the use of the antimonial and saline mixture, and of fomentations of opium and muriate of ammonia. These, with the exception of an occasional purgative, were the only medicines employed in the after-treatment. On the nineteenth day after the operation, Deyman left for home. The wound was nearly cicatrized, and all the urine came away through the natural passage. The vesical distress had subsided; and there was but little pain in urinating. The urine was sometimes high-colored, and contained a small quantity of sabulous matter. In all other respects the patient was entirely well.

The pencil, which was slit in half, the part containing the lead having gotten into the bladder, was two inches and five-eighths in length, and was conical at one extremity. It was coated with a deposit, which weighed about one drachm, and consisted of a small quantity of uric acid; the rest being triple phosphate.

I am indebted for full notes of this case to Dr. Henry Orton, of Canada West, and to my son, Dr. Samuel W. Gross, who both saw the patient frequently after the operation.

